# Hyocholic acid and glycemic regulation: *comments on ‘Hyocholic acid species improve glucose homeostasis through a distinct TGR5 and FXR signaling mechanism’*

**DOI:** 10.1093/jmcb/mjab027

**Published:** 2021-04-30

**Authors:** Wei Jia, Cynthia Rajani, Xiaojiao Zheng, Weiping Jia

**Affiliations:** 1 Center for Translational Medicine, Shanghai Key Laboratory of Diabetes Mellitus, Department of Endocrinology and Metabolism, Shanghai Jiao Tong University Affiliated Sixth People's Hospital, Shanghai Diabetes Institute, Shanghai 200233, China; 2 School of Chinese Medicine, Hong Kong Baptist University, Kowloon Tong, Hong Kong SAR, China; 3 University of Hawaii Cancer Center, Honolulu, HI 96813, USA

Hyocholic acid species (HCA, hyodeoxycholic acid, and their glycine and taurine conjugated forms) comprise 80% of the composition of pig bile (Haslewood, 1956). An interesting fact about pigs is that they do not get diabetes even though they eat almost everything and in abundant amounts, a diabetes-promoting diet. The first use of pig bile for treatment of ‘xiao-ke’, a condition known today as diabetes, was recorded ∼400 years ago by the Chinese medical practitioners in the *Compendium of Materia Medica* (Li, 1573‒1593). Recently, we found HCA species as novel biomarkers for metabolic diseases (Zheng et al., 2021b) and also identified the role of HCA species in the prevention of diabetes as well as their mechanism of action (Zheng et al., 2021a). Although bile acids (BAs) are mostly associated with their aid in food digestion, they have also been shown to act as signaling molecules by binding to two particular receptors, farnesoid X receptor (FXR) and the G-protein-coupled receptor TGR5. Experiments were thus directed to the effect of HCA binding to these two BA receptors on glycemic regulation in both *in vivo* and *in vitro* models.

The first *in vivo* experiment was done using pigs. Three groups of pigs were fed GW4064, an FXR agonist that caused significant suppression of HCA species production, along with 30% increase in blood glucose levels and 69% decrease in blood glucagon-like peptide-1 (GLP-1) levels. When HCA species were administered, the blood glucose levels decreased and circulating GLP-1 increased, suggesting that glucose homeostasis and GLP-1 secretion were regulated by HCA species. Further *in vivo* testing was then done in two diabetic mouse models. HCA species administration to the mice caused the most significant lowering of blood glucose and the most improved glucose tolerance results compared to metformin at a dose 2-fold higher than HCA and to tauroursodeoxycholic acid (TUDCA). Circulating GLP-1 levels were also significantly increased in the HCA group.

The BA receptors intestinal FXR and TGR5 are expressed in enteroendocrine L cells that are found primarily in the ileum and colon. Therefore, *in vitro* studies of the effects of HCA species were performed using the enteroendocrine L-cell lines, STC-1 and NCI-H716. Based on previous studies, which showed that BAs could induce GLP-1 release within 1–2 h (Thomas et al., 2009) and induce expression of the proglucagon gene within 24 h of treatment (Trabelsi et al., 2015), the effects of six different HCA species and six different non-HCA BAs on GLP-1 secretion and proglucagon gene expression were measured. At low BA concentration (5 µM), there was no increase in GLP-1 secretion or production, while at higher concentration (25 µM), all of the HCA species and the TGR5 agonists, lithocholic acid (LCA) and deoxycholic acid (DCA), stimulated GLP-1 secretion within 1 h and after 24 h. HCA species, TGR5 agonists LCA and DCA, and FXR antagonists TUDCA and tauro-β-muricholic acid promoted the transcription of proglucagon and GLP-1 secretion. At 50 µM concentration, HCA species surpassed all other BAs in the ability to increase proglucagon transcription and GLP-1 secretion. Similar results were achieved when human colonic explants were treated with the various BAs at 50 µM.

The next set of *in vitro* experiments were designed to measure direct effects of HCA species on the receptors by measuring intracellular cyclic adenosine monophosphate (cAMP) accumulation mediated by activation of TGR5 and also the effects of FXR agonists and HCA species (FXR antagonist) on the expression of the downstream FXR target, small heterodimer partner (SHP), and on GLP-1 secretion. These experiments are further discussed and diagrammatically illustrated in Figure 1. The necessity for TGR5 activation for increased secretion and production of GLP-1 was then confirmed *in vivo* by comparing the effects of HCA species administrated to TGR5^–/–^ and TGR5^+/+^ mice. Finally, a cohort of 55 participants comprised of 30 healthy, 18 pre-diabetic, and 17 newly diagnosed diabetic individuals were given an oral glucose tolerance test. Results revealed that GLP-1 secretion was much higher in the healthy group and that HCA species were inversely correlated with fasting and post-glucose load levels.

**Figure 1:**
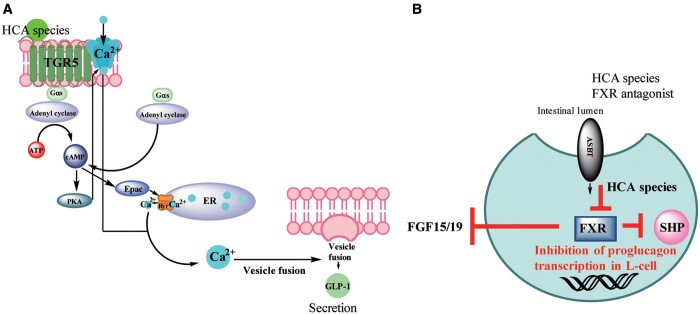
The effects of BA-activated TGR5 signaling and BA-inhibited FXR signaling in enteroendocrine L cells. L cells produce and secrete important hormones that affect energy metabolism and preserve pancreatic β-cell function. (**A**) In L cells, TGR5 is coupled to Gαs G-proteins. HCA species are found to be an agonist for TGR5 and act to promote the secretion of GLP-1, an incretin that has important effects on glucose homeostasis. Gαs protein coupling to BA-activated TGR5 results in the recruitment of adenyl cyclase, which subsequently activates cAMP to increase intracellular Ca^2+^ via the protein kinase A (PKA) or guanine nucleotide exchange factor (Epac) pathway and ultimately increases the secretion of GLP-1. An assay was performed, which detected increased production of cAMP upon treatment with HCA species, thus indicating that HCA species were the agonist for TGR5 (Zheng et al., 2021a). (**B**) HCA species are shown to be the L-cell FXR antagonist by their ability to reverse the inhibition of proglucagon transcription that leads to decreased GLP-1 production and secretion and also by being able to downregulate the expression of SHP, a downstream target of FXR. CDCA, an FXR agonist, gave opposite effects (Zheng et al., 2021a). ASBT, apical sodium-dependent bile acid transporter; ATP, adenosine triphosphate; ER, endoplasmic reticulum; FGF15/19, fibroblast growth factor 15/19; RYR, ryanodine receptor.

Although strong evidence was presented for the mechanism of action of HCA species in preventing or ameliorating diabetes, there are many unanswered questions that remain. The first fundamental question is how pigs developed the capability of producing HCA species in such large quantities. Previous studies have implicated gut microbiota such as *Ruminococcus productis* together with an unknown gram-positive rod called hyodeoxycholic acid-1 (HDCA-1) in the production of HCA species via bacterial biotransformation of β-muricholic acid (Eyssen et al., 1999). Other routes of HCA species biosynthesis include synthesis from non-12-hydroxylated BAs, LCA, taurolithocholic acid, and chenodeoxycholic acid (CDCA), via CYP3A4-mediated 6α-hydroxylation (Deo and Bandiera, 2008a; Jia et al., 2021) and conversion of LCA to 3α,6β-dihydroxy cholanoic acid, which then becomes further oxidized followed by reduction to become HDCA via gut microbiota (Deo and Bandiera, 2008b). Due to the connection between production of HCA species and the gut microbiota, one could hypothesize that differences in the composition of gut microbiota between pigs and humans may be part of the reason for their large difference in BA composition. Why does not the human body try to compensate and produce more HCA species in response to a diabetogenic diet given the strong relationship between diet and gut microbiota composition? If HCA species were administered at high doses for a prolonged period, would there be a change in the composition of the gut microbiota toward that of the pig? As most of the L cells are located in the ileum, would the ileal microbiota composition be the most affected by prolonged administration of HCA? Are HCA species capable of producing harmful side effects in humans/mice after prolonged exposure? Further studies are needed to assess any long-term side effects and length of efficacy for these unique BAs.

## References

[mjab027-B1] Deo A.K., Bandiera S.M. (2008a). Biotransformation of lithocholic acid by rat hepatic microsomes: metabolite analysis by liquid chromatography/mass spectrometry. Drug Metab. Dispos. 36, 442–451. 18039809 10.1124/dmd.107.017533

[mjab027-B2] Deo A.K., Bandiera S.M. (2008b). Identification of human hepatic cytochrome p450 enzymes involved in the biotransformation of cholic and chenodeoxycholic acid. Drug Metab. Dispos. 36, 1983–1991. 18583509 10.1124/dmd.108.022194

[mjab027-B3] Eyssen H.J., De Pauw G., Van Eldere J. (1999). Formation of hyodeoxycholic acid from muricholic acid and hyocholic acid by an unidentified gram-positive rod termed HDCA-1 isolated from rat intestinal microflora. Appl. Environ. Microbiol. 65, 3158–3163. 10388717 10.1128/aem.65.7.3158-3163.1999 PMC91470

[mjab027-B4] Haslewood G.A. (1956). Comparative studies of bile salts. 9. The isolation and chemistry of hyocholic acid. Biochem. J. 62, 637–645. 13315227 10.1042/bj0620637 PMC1215975

[mjab027-B5] Jia W., Wei M., Rajani C., et al (2021). Targeting the alternative bile acid synthetic pathway for metabolic diseases. Protein Cell *12*, 411–425. 33252713 10.1007/s13238-020-00804-9 PMC8106556

[mjab027-B6] Li S. (1573‒1593). Compendium of Materia Medica.

[mjab027-B7] Thomas C., Gioiello A., Noriega L., et al (2009). TGR5-mediated bile acid sensing controls glucose homeostasis. Cell Metab. 10, 167–177. 19723493 10.1016/j.cmet.2009.08.001 PMC2739652

[mjab027-B8] Trabelsi M.S., Daoudi M., Prawitt J., et al (2015). Farnesoid X receptor inhibits glucagon-like peptide-1 production by enteroendocrine L cells. Nat. Commun. 6, 7629. 26134028 10.1038/ncomms8629 PMC4579574

[mjab027-B9] Zheng X., Chen T., Jiang R., et al (2021a). Hyocholic acid species improve glucose homeostasis through a distinct TGR5 and FXR signaling mechanism. Cell Metab. 33, 791–803.e7. 33338411 10.1016/j.cmet.2020.11.017

[mjab027-B10] Zheng X., Chen T., Zhao A., et al (2021b). Hyocholic acid species as novel biomarkers for metabolic disorders. Nat. Commun. 12, 1487. 33674561 10.1038/s41467-021-21744-w PMC7935989

